# Elevated Serum TNF-*α* Is Related to Obesity in Type 2 Diabetes Mellitus and Is Associated with Glycemic Control and Insulin Resistance

**DOI:** 10.1155/2020/5076858

**Published:** 2020-01-30

**Authors:** Hana Alzamil

**Affiliations:** Physiology Department, College of Medicine, King Saud University, Riyadh, Saudi Arabia

## Abstract

**Background:**

Diabetes and obesity are very common associated metabolic disorders that are linked to chronic inflammation. Leptin is one of the important adipokines released from adipocytes, and its level increases with increasing body mass index (BMI). Tumor necrosis factor alpha (TNF-*α*) is a cytokine that is released by adipocytes and inflammatory cells in response to chronic inflammation. Type 2 diabetes mellitus (T2DM) is believed to be associated with low-grade chronic inflammation. The current study aims to investigate the involvement of leptin and TNF-*α*) is a cytokine that is released by adipocytes and inflammatory cells in response to chronic inflammation. Type 2 diabetes mellitus (T2DM) is believed to be associated with low-grade chronic inflammation. The current study aims to investigate the involvement of leptin and TNF-*Methodology*. This is a cross-sectional study involving 63 healthy volunteers and 65 patients with T2DM. Body composition was measured, and fasting venous blood samples were analyzed for blood glucose, glycosylated hemoglobin (HbA1c), basal insulin, leptin, and TNF-*α*) is a cytokine that is released by adipocytes and inflammatory cells in response to chronic inflammation. Type 2 diabetes mellitus (T2DM) is believed to be associated with low-grade chronic inflammation. The current study aims to investigate the involvement of leptin and TNF-*α*) is a cytokine that is released by adipocytes and inflammatory cells in response to chronic inflammation. Type 2 diabetes mellitus (T2DM) is believed to be associated with low-grade chronic inflammation. The current study aims to investigate the involvement of leptin and TNF-

**Results:**

Our study showed a significantly higher level of TNF-*α*) is a cytokine that is released by adipocytes and inflammatory cells in response to chronic inflammation. Type 2 diabetes mellitus (T2DM) is believed to be associated with low-grade chronic inflammation. The current study aims to investigate the involvement of leptin and TNF-*p*=0.008). In obese diabetic patients, the serum level of TNF-*α*) is a cytokine that is released by adipocytes and inflammatory cells in response to chronic inflammation. Type 2 diabetes mellitus (T2DM) is believed to be associated with low-grade chronic inflammation. The current study aims to investigate the involvement of leptin and TNF-*p*=0.008). In obese diabetic patients, the serum level of TNF-*p*=0.008). In obese diabetic patients, the serum level of TNF-*α*) is a cytokine that is released by adipocytes and inflammatory cells in response to chronic inflammation. Type 2 diabetes mellitus (T2DM) is believed to be associated with low-grade chronic inflammation. The current study aims to investigate the involvement of leptin and TNF-*r* = 0.361, *p*=0.008). In obese diabetic patients, the serum level of TNF-*r* = 0.361, *p*=0.008). In obese diabetic patients, the serum level of TNF-

**Conclusion:**

TNF-*α* is associated with concurrent obesity and T2DM and correlates with HbA1c. This suggests that TNF-*α* needs further investigation to explore if it has a role in monitoring the effectiveness of management in individuals with obesity and T2DM.*α*) is a cytokine that is released by adipocytes and inflammatory cells in response to chronic inflammation. Type 2 diabetes mellitus (T2DM) is believed to be associated with low-grade chronic inflammation. The current study aims to investigate the involvement of leptin and TNF-*α*) is a cytokine that is released by adipocytes and inflammatory cells in response to chronic inflammation. Type 2 diabetes mellitus (T2DM) is believed to be associated with low-grade chronic inflammation. The current study aims to investigate the involvement of leptin and TNF-

## 1. Introduction

Among the most fundamental systems required for survival are metabolic and immune systems, and there is a strong integration between regulation of metabolism and the immune responses. Chronic inflammation due to abnormal productions of cytokines and activation of inflammatory signaling pathways is closely associated with metabolic disorders such as obesity, insulin resistance, and T2DM [1]. Obesity is associated with increased plasma level of leptin, a protein that is released from adipose tissue to regulate body weight by decreasing appetite besides increasing metabolism and energy expenditure [2]. Adipose tissue is a metabolically active organ which in obese individuals can be the source of low-grade chronic inflammation. The inflammatory process plays an important role in pathogenesis of T2DM, and chronic inflammation precedes the onset of the disease [3].

The first proinflammatory cytokine recognized for its involvement in pathogenesis of insulin resistance and T2DM was TNF-*α*. It has been reported that TNF-*α* reduces the expression of insulin-regulated glucose transporter type 4 (GLUT4) which is located mainly in adipocytes and skeletal and cardiac muscles [4]. Moreover, TNF-*α* by inducing serine phosphorylation of insulin receptor substrate-1 can act as an inhibitor of peripheral insulin action which leads to insulin resistance [4]. Two recent meta-analyses indicated that both patients with type 1 and type 2 DM have significantly elevated levels of serum TNF-*α* which showed a positive correlation with insulin resistance [5, 6]. In obese T2DM patients, the TNF-*α* plasma level is related to the amount of visceral fat and is not instantly affected in poorly controlled diabetic patients by acute lowering of blood glucose level [7].

Research about the role of TNF-alpha in obesity and diabetes is not settled yet and is still a subject of active research work. Prior studies showed conflicting results about the correlation of obesity with TNF-alpha level. A recent systematic review and meta-analysis investigated the association between TNF-alpha and type 2 diabetes mellitus and concluded that there is considerable heterogeneity between studies and further work is needed [6]. Additionally, most of the previous studies included subjects with BMI <30 (i.e., overweight but not strictly obese).

In this study, we aim to look into the correlation between TNF-alpha, insulin resistance, and HBA1c level in addition to the association between TNF-alpha, obesity, and diabetes. Specifically, we will investigate the effect of obesity and diabetes separately on the level of TNF-alpha, and we aim to recruit participants with BMI higher than 30 kg/m^2^.

## 2. Methodology

In this cross-sectional study, we recruited healthy employees and patients with T2DM from the primary care clinics at King Khalid University Hospital, Riyadh, Saudi Arabia. The control group was evaluated by detailed history, clinical examination, and investigations. Patients with complications of T2DM such as nephropathy, neuropathy, and cardiovascular incidents were excluded from the study. All T2DM patients included in the study were receiving oral hypoglycemic agents, and 18 (24%) patients were on lipid lowering agents. Any patient with pregnancy and using glucocorticoids or oral contraceptive pills was excluded.

Body composition was measured using the body composition analyzer (Biospace-InBody 3.0. SNBS 300504E 2003/04.272-Iyongieong-vi, yipjang-myeon, chanan-si, chungcheongnam-do, South Korea). The following measurements were taken for all subjects: body mass index (BMI), percentage of body fat (BF%), lean body mass, and waist-hip ratio (WHR). Before those measurements were taken, the subjects were advised to fast for 10 hours and allowed to rest for 30 minutes. Palms and soles were cleaned with electrolytes tissue, and information about subjects' height, sex, and age was fed to the machine. The subject was asked to stand with barefoot on the platform of the machine.

Fasting venous blood samples were analyzed for blood glucose, glycosylated hemoglobin (HbA1c), basal insulin, leptin, and tumor necrosis factor alpha (TNF). HbA1c was measured by the Helena Glyco-Tek Affinity Column method (Helena Biosciences, Europe, Colima Avenue, Sunderland Enterprise Park, Sunderland, Tyne and Wear, SR53 × B, UK).

Insulin, leptin, and TNF-*α* immunoassays were performed by the quantitative standard sandwich ELISA technique using monoclonal antibody specific for these parameters with kits supplied by R&D Systems (Abingdon, United Kingdom). The indices of basal insulin resistance and beta-cell function were assessed using the homeostasis model assessment (HOMA-IR and HOMA-B) in which HOMA-IR (mmol/L × *μ*IU/mL) = fasting glucose (mmol/L) × fasting insulin (*μ*IU/mL)/22.5 and HOMA-B = fasting insulin (*μ*IU/mL × 20)/(fasting glucose (mmol/L) − 3.5).

## 3. Statistical Analysis

The data were analyzed by the computer software program Statistical Package for Social Sciences (SPSS version 20, Chicago). Descriptive characteristics of the study patients were expressed as mean ± SD (standard deviation). Kolmogorov–Smirnov^a^ and Shapiro–Wilk tests were used to see whether data follow normal distribution or not. Those parameters which were not following normal distribution were analyzed by nonparametric Mann–Whitney *U* test. For continuous data with normal distribution, Student's *t*-test was used. Correlation between TNF-*α*, HbA1c, and markers of insulin resistance was determined by simple regression analysis.

## 4. Ethics

The study was approved by the ethical committee of institutional review board of College of Medicine, and it was conducted at the Department of Physiology, College of Medicine, King Saud University, Riyadh. All participants signed a consent form after the study protocol was explained to them, and it was made clear to all subjects that they can withdraw at any time if they want to do so.

## 5. Results

One hundred twenty-eight subjects participated in this study: sixty-five subjects were diabetic patients (36 males and 27 females) and sixty-three were healthy control subjects (34 males and 31 females). Clinical and demographic characteristics were compared between controls and T2DM patients (Table 1). Age ranges for controls were 25–62 years (mean: 47.2 ± 7.7) and 30–66 years for diabetic patients (49.5 ± 10.2). The body mass index was significantly higher in patients with T2DM compared to controls (31.4 ± 5.7 and 28.9 ± 4.2, respectively; *p*=0.005). Patients with T2DM have a higher value of waist to hip ratio when compared to healthy controls (1.03 ± 0.08 and 0.97 ± 0.07, respectively; *p*=0.001). TNF-*α* level was significantly higher in T2DM patients than in controls (7.5 ± 2.48 and 6.2 ± 3.0, respectively; *p*=0.008), while the difference was not significant for leptin (32.2 ± 19 .5 and 30.6 ± 19.8, respectively; *p*=0.331) (Table 1).

**Figure 1:**
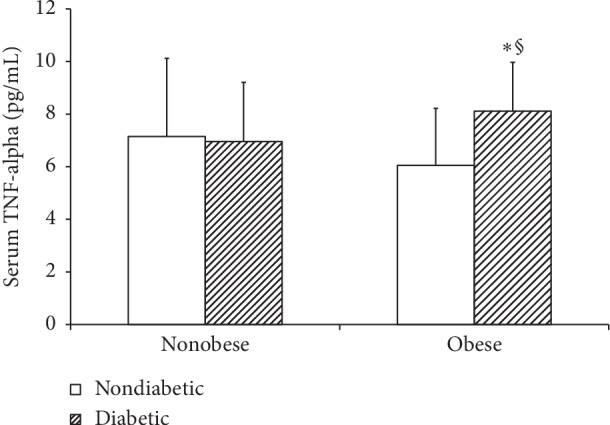
Effect of BMI on serum TNF-*α* levels (mean ± SEM). Nonobese nondiabetic group vs obese nondiabetic group; *p* value = NS. §Obese diabetic patients vs nonobese diabetic patients (*p* < 0.018). Nonobese nondiabetic group vs nonobese diabetic patients; *p* value = NS. ^*∗*^Obese diabetic patients vs obese nondiabetic group (*p* < 0.001). NS: not significant.

Serum TNF-*α* levels in obese diabetic patients were significantly higher than in nonobese diabetic patients (*p* < 0.018). The obese diabetic patients have significant higher serum TNF-*α* levels than the obese nondiabetic group (*p* < 0.001) as shown in Figure 1.

**Figure 2:**
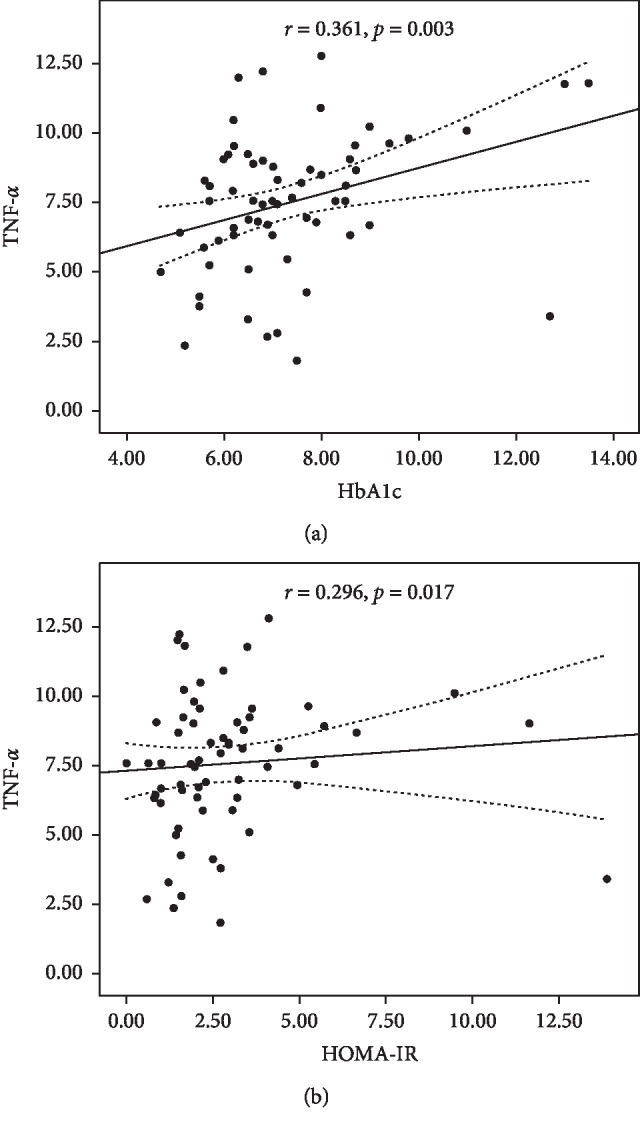
Correlation between serum TNF-*α* levels pg/ml (mean ± SEM) with (a) HbA1c and (b) HOMA-IR in diabetic patients (*p* < 0.003 and *p* < 0.017, respectively).

In patients with T2DM, the level of TNF-*α* correlated positively with HbA1c (*r* = 0.361, *p*=0.003) and HOMA-IR (*r* = 0.296, *p*=0.017), indicating a significant relationship with glycemic control and insulin resistance (Figure 2).

## 6. Discussion

Our study demonstrated that the serum TNF-*α* level was significantly higher in T2DM patients compared to healthy subjects while the difference between the two groups was not significant for leptin. The level of TNF-*α* has a strong positive correlation with HbA1c and was positively associated with insulin resistance. These findings suggest that TNF-*α* plays an important role in the pathogenesis of T2DM via mechanisms related to insulin peripheral action independent of leptin. Our patients have higher insulin resistance indices and a lower beta-cell function than control subjects.

The catabolic effect of TNF-*α* on adipose tissue and increased peripheral glucose uptake after neutralizing TNF-*α* in obese rats indicate its important role in development of insulin resistance and diabetes as a consequence of obesity [8]. Previous studies reported that TNF-*α* contributes to insulin resistance and T2DM that is associated with obesity [9–13]; however, Miyazaki et al. did not confirm this association [14]. In our study, we found a significantly higher TNF-*α* levels in obese diabetic patients compared to nonobese diabetic patients. Additionally, we reported a higher TNF-*α* level in obese diabetic patients in comparison with obese nondiabetic healthy subjects.

The majority of diabetic patients in the current study have BMI above 30, and their elevated TNF-*α* levels showed a significant positive correlation with insulin resistance. On the other hand, leptin level was not higher in our patients than in control subjects and its correlation with insulin resistance is weak. Owecki and coworkers performed a linear regression test for BMI and leptin to find the threshold of BMI at which serum leptin level starts increasing, and they reported a sudden increase in leptin levels at a BMI of 24.6 kg/m^2^ which indicates that some metabolic changes occur before reaching obesity levels [2]. These findings might explain why in our study we could not find difference in leptin level between diabetic patients and healthy controls since our control's BMI (28.9) was way above the threshold level.

We found that in patients with T2DM, the higher the level of TNF-*α*, the higher their HbA1c value, which means that TNF-*α* level can be used to predict glycemic control in obese diabetic patients. Mirza et al. showed that diabetes was strongly associated with elevated levels of TNF-*α*, which was most significantly elevated in the group of patients with HbA1c values higher than 6.5% [15]. In our study, the average value for HbA1c in patients with T2DM was 7.3% and it was positively associated with TNF-*α* level. Since HbA1c is not a direct measure of glycemia, there is a chance that its level might change due to factors unrelated to blood glucose levels such as rate of glycation and turnover of erythrocytes [16]. Recently, a meta-analysis of 19 studies indicated that elevated levels of inflammatory cytokines, including TNF-*α*, were strongly associated with increased risk to occurrence of T2DM [6]. Moreover, the finding that TNF-*α* level decreases with good glycemic control would confirm this hypothesis. Interestingly, a previous study showed a significant reduction in TNF-*α* serum level in obese T2DM patients following 4 weeks of treatment with diet and exercise [9]. Tsukui et al. reported a correlation between the drop in serum TNF-*α* level and HbA1c value after regular exercise in healthy women [17]. Theoretically, this association between TNF-*α* and HbA1c might be useful clinically to follow up the level of glycemic control in patients with T2DM after medical treatment or changed lifestyle or in cases where HbA1c cannot be trusted due to defect in glycation or severe anemia.

*Limitations*. This is a cross-sectional study with a limited number of patients. A longitudinal study with categorization of patients based on HbA1c and BMI would help in confirming our findings. Follow-up studies after good glycemic control to measure the improvement in beta-cell function and its association with decreased TNF-*α* level are our future plan.

## 7. Conclusion

Independent of leptin level, serum level of TNF-*α* in diabetic patients correlates with the level of insulin resistance and the glucose metabolism parameters such as HbA1c. The increased TNF-*α* level is related to the combined effect of obesity and diabetes while increased leptin level is caused by obesity unescorted by diabetes. Our findings suggest that further research should investigate the role of TNF-*α* as a tool to monitor the effectiveness of diabetes management, particularly in individuals with obesity.

## Figures and Tables

**Table 1:** Comparison of clinical characteristics, body composition, and insulin resistance indices between control and type 2 DM patients.

Variables	Control	DM	*p* value
M/F	36/27	34/31	
Age (years)	47.2 ± 7.7	49.5 ± 10.2	0.790
BMI	28.9 ± 4.2	31.4 ± 5.7	0.005
WHR	0.97 ± 0.07	1.03 ± 0.08	0.001
FBG (mmol/L)	5.0 ± 0.5	7.9 ± 2.6	0.001
HbA1c (%)	—	7.3 ± 1.8	—
Fat mass (kg)	26.6 ± 8.5	30.2 ± 10.6	0.040
BF%	34.9 ± 8.2	37.6 ± 7.4	0.032
Basal insulin (*μ*IU/ml)	6.5 ± 3.3	10.5 ± 14.4	0.028
HOMA-IR	1.5 ± 0.8	2.9 ± 2.4	0.010
HOMA-B (%)	95.5 ± 61.3	48.6 ± 34.5	0.001
TNF-*α* (pg/ml)	6.19 ± 3.01	7.51 ± 2.48	0.008
Leptin (ng/ml)	30.6 ± 19.8	32.2 ± 19 .5	0.331

M: males; F: females; BMI: body mass index; WHR: waist/hip ratio; FBG: fasting blood glucose; HbA1c: glycosylated hemoglobin; BF%: body fat percentage; HOMA-IR: homeostasis model assessment of insulin resistance; HOMA-B: homeostasis model assessment of beta-cell function; TNF-*α*: tumor necrosis factor alpha. Values are expressed as mean ± SD. Insulin and leptin levels were compared by the Mann–Whitney *U* test. All other parameters were compared by the *t* test.

## Data Availability

Data are available as excel file if requested.
